# An Information-Theoretic Framework for Evaluating Edge Bundling Visualization

**DOI:** 10.3390/e20090625

**Published:** 2018-08-21

**Authors:** Jieting Wu, Feiyu Zhu, Xin Liu, Hongfeng Yu

**Affiliations:** Department of Computer Science & Engineering, University of Nebraska-Lincoln, 1400 R St, Lincoln, NE 68588, USA

**Keywords:** information visualization, graph visualization, edge bundling, information theory, minimum description length

## Abstract

Edge bundling is a promising graph visualization approach to simplifying the visual result of a graph drawing. Plenty of edge bundling methods have been developed to generate diverse graph layouts. However, it is difficult to defend an edge bundling method with its resulting layout against other edge bundling methods as a clear theoretic evaluation framework is absent in the literature. In this paper, we propose an information-theoretic framework to evaluate the visual results of edge bundling techniques. We first illustrate the advantage of edge bundling visualizations for large graphs, and pinpoint the ambiguity resulting from drawing results. Second, we define and quantify the amount of information delivered by edge bundling visualization from the underlying network using information theory. Third, we propose a new algorithm to evaluate the resulting layouts of edge bundling using the amount of the mutual information between a raw network dataset and its edge bundling visualization. Comparison examples based on the proposed framework between different edge bundling techniques are presented.

## 1. Introduction

Graphs, also known as networks, are used to represent interrelated objects, where the objects of a graph are denoted as nodes or vertices, and the relationships among the objects are denoted as links or edges. Node–link diagram is one of the most commonly used graph presentations that depicts a 2D or 3D graph drawing where vertices are visually presented as dots or labels, and links are visually presented as segments, polylines, or curves. Node–link diagram is widely used in diverse areas because of its intuitiveness and simplicity. However, segment-based node–link diagram starts to lose its effectiveness with the increasing size of graph, because excessive edge crossing and overlapping are incurred, sometimes even with hundreds of edges. Edge bundling can effectively reduce visual clutter and help better reveal the structure and patterns in complex networks. The basic idea of edge bundling is to group similar edges and visually present them using polylines or spline curves. The final graph drawing consists of several subgraphs that are pictorially presented by bundles. It can reduce small-angle edge crossings and total drawing area used. The layout of the graph drawing is thus simplified and becomes easier for comprehension.

Researchers and designers have devoted many efforts to developing various edge bundling techniques. However, few studies provided a clear theoretic framework to evaluate the existing edge bundling methods. Although readability metrics [1,2,3,4,5] are widely acknowledged to evaluate graph drawings, they mainly focus on segment-based node–link diagrams. A faithfulness metric [6] was recently introduced to measure the quality of a graph drawing mapping from its underlying network structure to its visual presentation. It is merely a semi-formal model. The literature still lacks a convincing framework to assess edge bundling drawings. Without a proper evaluation method, it is not easy for domain users to choose appropriate edge bundling methods with proper parameters for their applications. Meanwhile, it is difficult for visualization researchers and designers to defend the quality of their edge bundling methods. These challenges are also enunciated in the recent edge bundling reviews [7]. A theoretic assessment framework is thus critical for the development and deployment of edge bundling techniques.

We propose an information-theoretic framework to evaluate edge bundling techniques based on information theory. We leverage the framework proposed by Chen et al. [8,9] to elucidate the visualization process of edge bundling visualization. First, we discuss that edge bundling techniques are advantageous for visualizing a large graph (e.g., with thousands or millions of edges) that is hard to be effectively tackled by segment-based node–link diagram. Second, we argue that the criteria to evaluate edge bundling visualizations can be defined by finding the actual number of paths between pairs of vertices in the corresponding edge bundling drawing. We use conventional image processing algorithms, region growing and mean shift, to computationally find the actual number of paths between a pair of vertices in edge bundling drawings. Finally, our theoretic approach quantifies the amount of uncertainty of edge bundling algorithms based on the mutual information between a raw network dataset and its corresponding visualization. We conclude that a good edge bundling result should maximize the amount of mutual information based on information theory. We present several examples of comparison between three representative edge bundling algorithms, and show that our approach can provide a convincing assessment to the existing edge bundling visualizations.

## 2. Related Work

### 2.1. Graph Visualization and Evaluation Metrics

Graph visualization can help reveal the structure and patterns of an underlying network, and has been applied in various scientific and engineering domains, such as biological, technological, and social systems. The information visualization community has developed many sophisticated graph visualization methods. These methods mostly employ matrix, node–link diagram, and hybrid visual presentations, which help researchers and domain experts effectively gain insights into their data. A wide spectrum of graph visualization techniques and their applications have been surveyed in many studies. We refer readers to the following works and surveys for the diverse graph visualization approaches. Landesberger et al. [10] and Beck et al. [11] reviewed techniques for visualizing large graphs and dynamic graphs respectively. Herman et al. [12] and Vehlow et al. [13] conducted comprehensive surveys for graph visualization. Wang et al. [14] presented a visual analytics framework to reveal the ambiguity of graph drawings. To evaluate the quality of graph drawings, researchers have provided and discussed many metrics for graph readability [1,2,3,4,5,15,16,17,18,19,20] and graph faithfulness [6]. The readability of a graph drawing is often measured by aesthetics criteria. They can be concluded as to minimize edge crossings, edge bends, and area used, and maximize orthogonality and structure symmetry. To optimize two or more of these criteria is NP-hard [21]. Among the criteria, edge crossing is widely acknowledged as the most important one [2]. Edge bends, orthogonality, and structure symmetry are not practical to evaluate the quality of edge bundling drawings. Edge bundling methods inherently create many edge bends, which indeed reduce many small-angle edge crossing to avoid visual occlusion. Orthogonality and structure symmetry criteria often require adjusting the positions of vertices. In many real-world applications, the positions of vertices are immovable to show the geo-location information. Nguyan et al. [6] introduced faithfulness of graph drawings, which is an important criterion that measures the degree that a graph drawing algorithm can uniquely present the raw network data. Simply, if a graph drawing algorithm is faithful, it can map different graph drawing results to distinct networks. However, Nguyan et al. indicated that it is only a semi-formal model without a precise description. In this paper, we discuss the readability and faithfulness models and propose an information-theoretic framework to quantify the amount of information delivered by edge bundling visualization from original network data.

### 2.2. Edge Bundling Visualization

Edge bundling visualization has significant impacts on various scientific and engineering domains, such as life science [22,23,24], software engineering [25,26,27], social science [28,29], and so on. Lhuillier et al. [7] provided a comprehensive review on edge bundling methods. Holten [30] introduced a novel edge-clutter-reduction drawing algorithm for graphs that have their own hierarchical structures. The edges are bundled using B-splines, of which the control points are defined by the hierarchical structures. Several following works [29,31] also employed hierarchical structures as a guideline to organize edge bundles. Zhou [32] employed a force model to visualize high-dimensional and multivariate datasets in parallel coordinates. Holten et al. [33] further developed the force model in general graphs, namely, force-directed edge bundling (FDEB). A set of methods and applications [34,35,36] follow a similar idea. Some methods [37,38,39,40] use geometric control meshes to guide bundling process. Ink minimization methods [41,42] generate edge bundles using ink minimization metrics. Image-based methods [22,43,44,45,46,47] use density estimation to create edge bundles. Recently, piecewise polynomial basis functions are employed in edge bundling methods [48,49]. Although considerable efforts have been made to design new edge bundling methods, the comparisons among different methods mainly rely on their efficiency. Few studies focus on comparing the quality of the visual results of different methods. An evaluation [30] has been made to assess the hierarchical edge bundling method, but it is difficult to be generalized to other methods. Wu et al. [48] proposed a quality metric that uses a ratio of curve distortion to used pixel to evaluate the quality of the visual results of edge bundling algorithms. The proposed metric does indeed consider the readability criteria such as edge bends, edge crossings, and area used. However, it does not consider the faithfulness criteria. Therefore, it remains an open problem to quantify the quality of edge bundling algorithms.

### 2.3. Studies of Information Theory in Visualization and Computer Graphics

Information theory was first introduced by Shannon to illustrate communication systems [50,51]. Since then, information theory has been widely used in various fields. The information visualization and scientific visualization communities have been using information theory and its related concepts in many studies [8,52,53,54]. Chen et al. [8,9] adopted the model and presented an information-theoretic visualization system. Researchers have used entropy measures in viewpoint selection for time-varying volume rendering [55], vector field visualization [56], and isosurface rendering [57]. Measures of mutual information were used to select optimal viewpoints for 3D objects [58,59]. For a comprehensive survey of information-theoretic framework in scientific visualization, we refer readers to Wang et al.’s paper [54]. In computer graphics, an analysis of scene visibility and radiosity complexity was introduced by Feixas et al. [60]. Rigau et al. [61] leveraged the entropy of pixel color and geometry to guide pixel supersampling in stochastic raytracing. Fleishman et al. [62] proposed a viewpoint selection for modeling a 3D object from images. Gumhold [63] suggested a solution for light sources placement using information theory for image models. Vazquez et al. [64] used viewpoint entropy to select an optimal view angle for polygon models. We refer readers to Sbert et al.’s work [65] for more complete information of information theory in computer graphics. Since the concepts and details of information theory have been introduced and explained in many existing works [50,51,66,67,68,69,70], we only list the necessary mathematical formulas of information theory in this paper to make the content concise.

## 3. Method

### 3.1. Background

**Figure 1:**
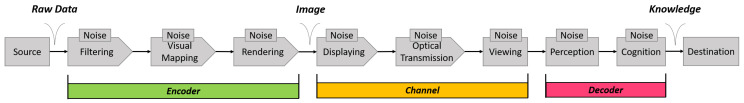
A general visualization pipeline.

Chen et al. [8,9] presented a comprehensive information-theoretic pipeline to illustrate the visualization process using information theory, as shown in Figure 1. This pipeline can be generally applied to graph visualization. The pipeline shows that a graph visualization first encodes raw data, and then sends the encoded visual description, e.g., a drawing (image) via a visual channel. An observer receives the visual description and tries to decode the description for final comprehension. The general visualization pipeline has been used in many existing work. Since the knowledge about the decoder (human perception and cognition) in Figure 1 may require a tremendous number of user studies, we are reluctant to conduct a comprehensive study that covers the full span of the visualization pipeline. Similar to the recent work [71], we only focus on the encoder subsystem. We discuss how the raw network data are encoded and described as an image by edge bundling algorithms and how to optimize the information transferring using Chen et al.’s pipeline and information theory.

**Figure 2:**
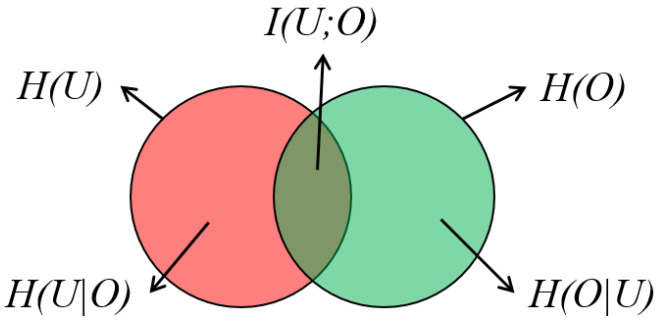
A Venn diagram showing the relation of entropies H(U) and H(O), and mutual information I(U;O).

In the encoder subsystem, the process of filtering, visual mapping, and rendering can be considered to be a transformation or encoding process, and a good graph visualization should use a visual description to tell as much information about the raw data as possible. Simply, we define the raw data as a random variable *U*, and consider the encoder to be an encoding process. To describe *U*, the encoding process may first use some simplification methods such as filtering and clustering to preprocess *U*, and then present the preprocessed data visually as labels, points, lines, or areas. The output of this process is a visual description *O*. Information may lose and noise may be introduced in this process. In many existing works, a good graph visualization is concluded to make *O* tell the most about *U*. To do that, the mutual information I(U;O) between the visual description and the raw data should be maximized. Formally,

(1) I(U;O)=H(U)−H(U|O),

where H(U) is the total information of *U*, and the conditional entropy H(U|O) is the amount of additional information needed to describe *U* given *O*. It can also be regarded as the information loss in the encoding process. To maximize the mutual information I(U;O), we thus need to minimize H(U|O), i.e., to minimize the information loss. Figure 2 illustrates H(U), H(O) and I(U;O), and their relationship. Many visualization studies [58,59,60,71,72,73,74,75,76,77] have proposed solutions to maximize the mutual information to improve their visualization results.

### 3.2. Uncertainty in Edge Bundling Visualizations

**Figure 3:**
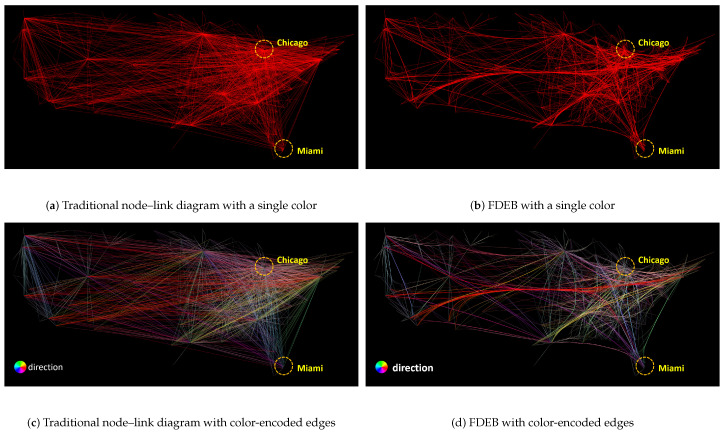
The comparison to find if *Chicago* and *Miami* are connected using: (**a**) traditional node–link diagram with a single color; (**b**) force-directed edge bundling visualization with a single color; (**c**) traditional node–link diagram with color-encoded edges; and (**d**) force-directed edge bundling visualization with color-encoded edges.

The pipeline of Figure 1 can also be applied in edge bundling visualizations. In many existing works, edge bundling algorithms are used to visualize large graphs since the algorithms help reduce visual clutter. We illustrate the advantage of edge bundling visualizations over traditional node–link diagrams in large graph visualizations. A classic example is to visualize the U.S. airline routes where domain experts want to see the airline routes between different cities with their geo-locations, as shown in Figure 3a. The dataset of Figure 3a has 2100 edges and 235 vertices. The drawing in Figure 3a shows a traditional node–link diagram that uses segment-based edges to encode the relations of the graph, resulting in severe visual clutter because of the edge crossings and edge overlapping. The visual clutter mainly affects human perception to track the edge between a pair of vertices. For example, observers cannot easily tell if *Miami* and *Chicago* have a connection based on the drawing.

Traditional node–link diagrams may fall short in visualizing large graphs since they do not meet some readability criteria. As mentioned in Section 2, there were several readability criteria to evaluate the quality of a graph drawing. To optimize two or more of the readability criteria is an NP-hard problem [21]. Among the criteria, edge crossing is widely acknowledged as the most important one [2]. In graph drawings, edge crossings would cause visual ambiguity to observers. The ambiguity mainly affects human perception to identify the relations between pairs of vertices in graphs. In Figure 3a, the area between *Miami* and *Chicago* are occupied by many edges. Observers can hardly identify if *Miami* and *Chicago* are connected because of the visual clutter. To reduce the clutter, edge bundling techniques are often employed in the visualizations of large graphs. Edge bundling techniques mainly group similar edges to form bundles, such that the area used and edge crossing are significantly reduced. Figure 3b shows a force-directed edge bundling (FDEB) drawing using the U.S. airline dataset. It becomes much easier to identify the edge between *Miami* and *Chicago* compared to Figure 3a. Additionally, using color-encoded methods can help better identify paths and structural patterns. Color-encoded methods can customize the transparency and color of edges in a drawing based on the attributes of the edges. Figure 3c shows a directional color-encoded method for the node–link diagram of the airline dataset. Comparing Figure 3a,c, the edges with different directions are more salient in Figure 3c, whereas the path between *Miami* and *Chicago* can still be hardly tracked. Using the same color-encoded method in the FDEB visualization of the same dataset, we can clearly see the blue path connecting *Miami* and *Chicago*, as shown in Figure 3d. The overall result of Figure 3d is even better than Figure 3b. Intuitively, we can conclude that color-encoded method can help track the relations between vertices in visualizations.

**Figure 4:**
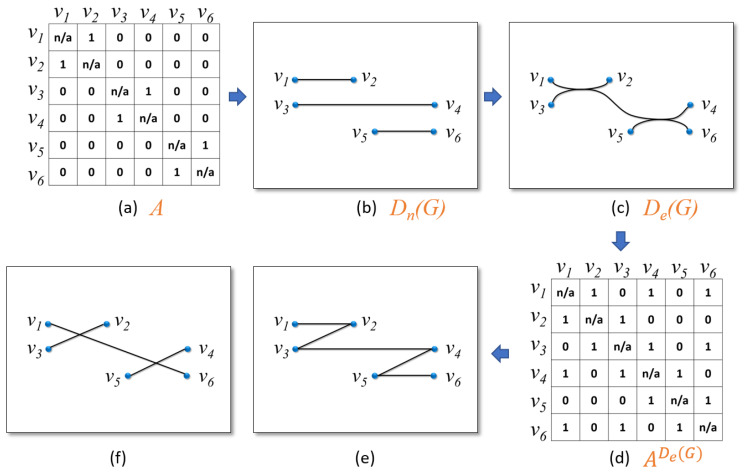
(**a**) An adjacency matrix *A* of a graph *G*. (**b**) A traditional node–link diagram $D_{n}\left(G\right)$ of *G*. (**c**) An edge bundling drawing $D_{e}\left(G\right)$ of *G* using curve presentation. (**d**) An adjacency matrix $A^{D_{e}\left(G\right)}$ interpreted from $D_{e}\left(G\right)$. (**e**,**f**) Two possible unbundled graph structures that are derived from $A^{D_{e}\left(G\right)}$ and can form the same edge bundling drawing in (**c**).

Although edge bundling techniques can improve the readability of graphs in terms of edge crossing and area used, they are not without disadvantages. Edge bundling methods visually create bundle effect to reduce edge crossings and area used, but the relationship details are thus hidden in the bundle. An example is shown in Figure 4. Figure 4c shows an edge bundling drawing. Figure 4b,e,f shows three possible network results that can generate the same edge bundling drawing in Figure 4c. This disadvantage is also discussed in several papers. Wang et al. [14] provided a visual analytic tool to show the ambiguous regions using heat map in graph drawings. They considered edge lengths, vertex and edge aggregations, and community structures; however, they did not consider the inter-bundle ambiguity, i.e., the uncertainty of two vertices from different bundles. Figure 4c illustrates an example about the inter-bundle condition. We can visually perceive that there are approximately two bundles in Figure 4c. Two examples of intra-bundle ambiguity are that the relation between vertices $v_{2}$ and $v_{3}$, and the relation between vertices $v_{4}$ and $v_{5}$ are unknown. Meanwhile, using the aforementioned method cannot identify the ambiguity between vertices $v_{1}$ and $v_{6}$ in the drawing, which corresponds to a case of inter-bundle ambiguity. Hence, we argue that the work is not sufficient to evaluate edge bundling drawings. Nguyen et al. [6] defined the uncertain presentation in an edge bundling visualization as information loss in the edge bundling visualization by introducing information faithfulness. A visualization is information faithful if the visualization can uniquely represent the original graph. In their paper, they concluded that edge bundling visualization is inherently not information faithful and stated that it will be increasingly difficult for users to perceive the original network from an edge bundling visualization when more edges are bundled together. They gave a model to illustrate this situation. Given a graph G=(V,E), the edge bundling visualization partitions the edges *E* into *K* bundles $E=B_{1}\cup \ldots \cup B_{i}\cup \ldots \cup B_{k}$. Let $G_{i}$ presents a subgraph of *G* that consists of only $B_{i}$. $G_{i}$ is essentially a bipartite graph where the set of vertices are $V_{i}$ and the set of links are $B_{i}$. For any two subgraphs $G_{i}$ and $G_{j}$, $B_{i}\cap B_{j}=\emptyset$. According to the definition of bipartite graph, we have $V_{i}=P_{i}\cup Q_{i}$, $P_{i}\cap Q_{i}=\emptyset$, where $P_{i}$ is the source vertices and $Q_{i}$ is the sink vertices in the bipartite graph $G_{i}$. Enumerating the bipartite graphs with all possibilities gives $2^{|P_{i}\left|\right|Q_{i}|}$ combinations. The number of graphs that have the same link structure as the final edge bundling drawing of *G* is $\prod_{i}^{k}2^{|P_{i}\left|\right|Q_{i}|}$, which means there are $\prod_{i}^{k}2^{|P_{i}\left|\right|Q_{i}|}$ different original networks. However, we argue the $2^{|P_{i}\left|\right|Q_{i}|}$ different ways that a bundle may have is just a loose upper bound. The tight bound requires further investigation. On the other hand, it is hard to define an exact number of bundles in a drawing. Additionally, their work also did not consider the inter-bundle uncertainty, as they assumed $B_{i}\cap B_{j}=\emptyset$, which often is not held in practice. Thereby, to evaluate the quality and goodness of edge bundling visualizations is still an open problem. In Section 3.3, we introduce a formal information-theoretic metric to evaluate the drawing result of edge bundling techniques.

### 3.3. An Information-Theoretic Metric for Edge Bundling Visualizations

We introduce a general model to quantify the uncertainty delivered by graph visualizations. We define the objects of a graph as nodes or vertices, and the relationships among objects as links or edges. Take a graph G=(V,E) where there are |V| vertices and |E| edges. In our study, we only consider simple paths in graph structures, and represent *G* as an adjacency matrix *A*:

(2) $$A_{ij}=\{\begin{array}{ll} 1 & \mathrm{there}\;\mathrm{is}\;\mathrm{an}\;\mathrm{edge}\;e_{ij}\;\mathrm{between}\;\mathrm{two}\;\mathrm{vertices}\;i\;\mathrm{and}\;j\\ n/a & i=j\\ 0 & \mathrm{otherwise} \end{array}.$$

Let D(G) denote a graph drawing of *G*, the edges and vertices of G are encoded by visual symbols (such as segments, curves, polylines, labels, points, etc.) with colors in D(G). Based on the pipeline of Figure 1, a graph drawing method has a visual encoding process that transforms the underlying network relations and structure into visual symbols. Color mapping functions are also used in the process. The encoder process outputs a visual description, i.e., D(G). Observers need to observe D(G) in order to guess the value of $A_{ij}$ of *G*. Visually, D(G) presents an adjacency matrix $A^{D(G)}$ indicating the relations among the vertices in the drawing:

(3) $$A_{ij}^{D(G)}=\{\begin{array}{ll} N(N\subset N) & \mathrm{there}\;\mathrm{are}\;N\;\mathrm{edges}\;\mathrm{between}\;\mathrm{two}\;\mathrm{vertices}\;i\;\mathrm{and}\;j\\ n/a & i=j\\ 0 & \mathrm{otherwise} \end{array}.$$

To understand the relations of the underlying network, observers need to observe D(G), and guess the value of $A_{ij}$ based on the value of $A_{ij}^{D(G)}$.

Figure 4 shows an example. Figure 4a shows an adjacency matrix *A* of a graph *G*. In Figure 4b, a node–link drawing $D_{n}\left(G\right)$ correctly reveals the relations among vertices with the least ambiguity for this simple graph. In Figure 4c, an edge bundling drawing $D_{e}\left(G\right)$ encodes the edges with curves. One observation is that there seems to be an edge between the vertices $v_{1}$ and $v_{2}$. One possible reason is that there indeed is an edge between $v_{1}$ and $v_{2}$. However, it is also possible that there is no edge between $v_{1}$ and $v_{2}$, but an edge between $v_{1}$ and $v_{6}$ and an edge between $v_{2}$ and $v_{3}$, and these two edges are bundled together, causing an illusion edge between $v_{1}$ and $v_{2}$. Hence, the ambiguity arises that the relation between $v_{1}$ and $v_{2}$ is uncertain in Figure 4c. Note that even if we only consider simple paths in the visualization of a graph *G*, $D_{e}\left(G\right)$ may inadvertently have multiple edges between a pair of vertices. By using certain intuitive criteria, e.g., readable bendiness of curves (Section 3.4), we can guess that there is no edge among $v_{1}$, $v_{3}$, and $v_{5}$ in $D_{e}\left(G\right)$. The same intuitiveness can be also applied to $v_{2}$, $v_{4}$, and $v_{6}$. However, all other relations among the vertices remain uncertain in $D_{e}\left(G\right)$. We can use Equation (3) to construct an adjacency matrix $A^{D_{e}\left(G\right)}$, as shown in Figure 4d. If an entry $A_{ij}^{D_{e}\left(G\right)}>0$, we are not sure if there would be an edge between *i* and *j* in the original graph *G*, and possibly drive multiple interpretations, such as Figure 4e,f, that can generate the same edge bundling draw in Figure 4c.

To assess an edge bundling drawing $D_{e}\left(G\right)$, based on *A* and $A^{D_{e}\left(G\right)}$, we first introduce a coverage rate λ to evaluate the percentage of how many edges in *A* are covered by $A^{D_{e}\left(G\right)}$. The idea is intuitive: we want to know how many edges in an original network are presented in a corresponding drawing. In our definition, we only require the drawing to show at least an edge between two vertices (i.e., $A_{ij}^{D_{e}\left(G\right)}>0$) if the two vertices do have an edge in the underlying network (i.e., $A_{ij}=1$). Equation (4) expresses this idea as (the **n/a** entries of *A* and $A^{D(G)}$ do not enter the computation of the following equations):

(4) $$\lambda =\frac{\sum_{i=1}^{m}\sum_{j=1}^{m}\mu (i,j)}{\left|E\right|},$$

where *m* is the number of vertices in *G*, and μ(i,j) is a simple Heaviside step function:

(5) $$\mu \left(i,j\right)=\{\begin{array}{ll} 1 & \mathrm{if}\;A_{ij}=1\;\mathrm{and}\;A_{ij}^{D_{e}\left(G\right)}>0\\ 0 & \mathrm{otherwise} \end{array}.$$

The higher the value of λ is, higher the coverage is. If λ=1, we say the corresponding $A^{D_{e}\left(G\right)}$ is saturated. However, only using Equation (4) cannot assess an edge bundling drawing effectively. For example, although Figure 4b,c covers the matrix *A* in Figure 4a, the degrees of uncertainty are significantly different in Figure 4b,c. Hence, we need to introduce another metric to evaluate the uncertainty of edge bundling drawing results.

We provide an information-theoretic model to quantify the uncertainty of the above situation. Our information-theoretic model first assumes that graph drawing and visualization algorithms do not intend to underdraw a graph, i.e., given a graph *G*, graph drawing and visualization algorithms do not intend to show a wrong value of $A_{ij}$ in $A_{ij}^{D(G)}$. If $A_{ij}=0$ in *G*, the encoder of visualization process should always intend to show $A_{ij}^{D(G)}=0$ in D(G), such that the observers may guess that $A_{ij}=0$ in *G*, and vice versa. In addition, if $A_{ij}^{D(G)}=N\left(N\subset N,N\geq 1\right)$ in D(G), it becomes more uncertain to determine whether $A_{ij}=1$ since *i* and *j* may overlap some edges that are not between *i* and *j* in *G*. Figure 4c shows an example. We know that even if only simple paths are allowed between a pair of vertices, a drawing result may still have multiple paths between this pair of vertices, which makes the visual description uncertain. We also define that the edge in Equation (2) is just a relation while the edge in Equation (3) can be a segment, curve, or polyline in the drawing.

We denote the relation between two vertices as a random variable *X*. A graph drawing or visualization algorithm fully understands the graph and tries to encode the relation with visual symbols and colors. After the encoding process, the algorithm outputs an image, i.e., a drawing D(G). D(G) provides a result that indicates the original relation of the two vertices. The visual result can be represented by an adjacency matrix $A^{D(G)}$ based on Equation (3). Given two vertices *i* and *j*, we can quantify the amount of uncertainty of the relation of vertex *i* and *j* given D(G) using information theory. Generally, if $A_{ij}^{D(G)}=N\left(N\subset N\right)$, there are *N* paths between the vertices *i* and *j* in the drawing D(G). Since we only consider simple paths, one of the *N* paths may be encoded as an edge to present $A_{ij}=1$ in *G*. Another possibility is $A_{ij}=0$, which means there is no edge between the vertices *i* and *j* in the original graph, but *i* and *j* overlap *N* other edges in the drawing D(G). Therefore, D(G) can have N+1 different ways to present $A_{ij}$ (i.e., *N* possible paths or no path).

Let *Y* denote the visual description of the relation between a pair of vertices. The amount of uncertainty of knowing the relation between the vertices *i* and *j* given D(G), i.e., the conditional entropy $H(X_{ij}|Y_{ij})$, can be quantified as:

(6) $$\begin{array}{cc} H(X_{ij}|Y_{ij}) & =\sum_{t=0}^{A_{ij}^{D(G)}}-\left(\frac{1}{A_{ij}^{D(G)}+1}\right)log_{2}\left(\frac{1}{A_{ij}^{D(G)}+1}\right)\\ & =log_{2}\left(A_{ij}^{D(G)}+1\right). \end{array}$$

where $A_{ij}^{D(G)}$ is the value of the *i* column and *j* row entry of $A^{D(G)}$. Equation (6) indicates the necessary bits to visually describe the relation of the corresponding vertices *i* and *j*. The more bit the visual description uses, the more uncertain the description is.

**Figure 5:**
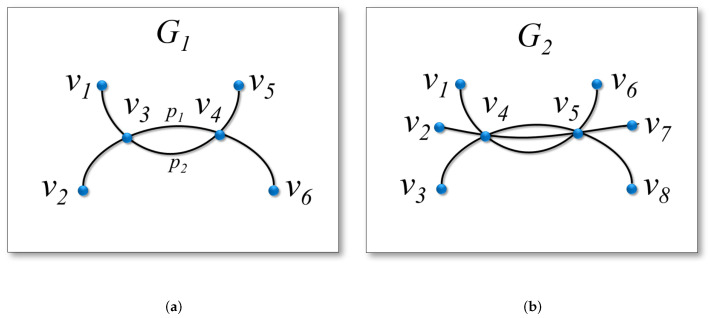
The graph drawings of two simple graphs $G_{1}$ and $G_{2}$. (**a**) A simple path graph $G_{1}$, where there are visually 2 paths between $v_{3}$ and $v_{4}$. (**b**) A simple path graph $G_{2}$, where there are visually 3 paths between $v_{4}$ and $v_{5}$.

We use the graph drawings of two simple graphs $G_{1}$ and $G_{2}$ in Figure 5 to illustrate Equation (6). As shown in Figure 5a, visually, there are two paths, $p_{1}$ and $p_{2}$, between the vertices $v_{3}$ and $v_{4}$, and thus $A_{3,4}^{D(G_{1})}=2$. As we consider simple paths, there are three possible cases of connection between $v_{3}$ and $v_{4}$ in the original graph $G_{1}$: $p_{1}$, $p_{2}$, or no path. The probability of each case is $\frac{1}{3}$. Therefore, given the visual description $Y_{3,4}$, we can compute the amount of uncertainty to describe the real relation $X_{3,4}$ as $\sum_{t=0}^{2}-(\frac{1}{3})log_{2}\left(\frac{1}{3}\right)=log_{2}\left(3\right)\approx 1.58$. Similarly, there are three paths between the vertices $v_{4}$ and $v_{5}$ of $G_{2}$ in its drawing $D(G_{2})$, as shown in Figure 5b. This leads to a higher amount of uncertainty, $\sum_{t=0}^{3}-(\frac{1}{4})log_{2}\left(\frac{1}{4}\right)=log_{2}\left(4\right)=2$, for us to tell the real relation $X_{4,5}$ of $G_{2}$.

We denote the total uncertainty of D(G) as *W*. H(W) can be formally written as:

(7) $$\begin{array}{c} H(W)=\sum_{i=1}^{m}\sum_{j=1}^{m}H(X_{ij}|Y_{ij}), \end{array}$$

where $H(X_{ij}|Y_{ij})$ represents the amount of uncertainty of knowing $X_{ij}$ based on $Y_{ij}$. It can also be interpreted as how much information about $X_{ij}$ is still uncertain after observing $Y_{ij}$. As discussed in Section 3.1, the best description *Y* should tell the most of *X* (i.e., to maximize the mutual information I(X;Y), we need to minimize the conditional entropy H(X|Y)). Hence, to holistically evaluate an edge bundling drawing, we argue that a good edge bundling visualization should minimize H(W), and, at the same time, keep the coverage rate λ as high as possible. For a undirected graph *G*, *A* and $A^{D(G)}$ are symmetric, and thus only the upper right half of each matrix is used.

We use Equation (7) to quantify the values of H(W) of Figure 4b,c. On one hand, as Figure 4b shows, the relations among objects are clear and correct, and the amount of uncertainty of the corresponding H(W) is 3. Figure 4b can use the least bits to describe a network since each edge can be distinguished in this drawing. On the other hand, the amount of uncertainty of H(W) of Figure 4c is 8. This comparison matches the results of the drawings.

Generally, Equation (6) can also be used to quantify the amount of uncertainty of relation between a pair of vertices in an edge bundling drawing D(G). Figure 3a shows a more complex example. In the figure, we want to quantify the amount of uncertainty of relation between *Miami* and *Chicago*. Equation (6) counts the number of edges (paths) between the cities in the drawing. Recall that the paths can be segments, curves, or polylines in our definition. We can find multiple such paths between *Miami* and *Chicago*. In Figure 3b, the paths between *Miami* and *Chicago* are significantly reduced because of the bundle effect. The rationale behind Equation (6) is that, if more paths can be detected between the two vertices, the used area between the two vertices becomes larger. This reflects that a larger number of edge crossings and overlapping, which means the visual description of the relation between the two vertices is more uncertain. The greater the value $A_{ij}^{D(G)}$, the more uncertain the description is. In a general case, no matter $A_{ij}=0$ or 1. If the number of paths between the two vertices is larger than one, i.e., $A_{ij}^{D(G)}>1$, the visual description of the relation of the two vertices is uncertain. In Section 3.4, we introduce a method to count the number of paths between a pair of vertices based on the drawing.

### 3.4. Algorithms and Implementation

We introduce an algorithm to approximate the number of edges or paths between a pair of vertices in a drawing. Our algorithm mimics an observer’s perception to track the edges or paths between two vertices. As it would be tedious for an observer to manually count all paths in a drawing of a large graph, we propose a computational method for this task. We also use a heuristic method to discuss the parameters in our algorithm in Section 4. As described in Section 3.3, a path between two vertices in a drawing can be segments, spline-curve, polylines, or a hybrid presentation of the above three. Generally, a qualified path between two vertices should meet two criteria: (1) the bendiness of the path should be reasonable; and (2) the color of the path should be similar. The bendiness criteria ensures that a qualified path have a reasonable smoothness and do not contain loops and abrupt turning angles, while the color criteria ensures that the color along the path is similar. Complying the two criteria, a path can be identified and tracked by observers. Although many studies have proposed path and road location and detection in remote sensing and image processing fields, approximating the number of paths between two vertices is a unique and non-trivial task in this study.

To approximate the number of qualified paths between two vertices, we need to find the region in the image connecting them. We first locate the pixel positions of a pair of vertices in an edge bundling drawing (image). Then, starting from one of the vertices, we conduct a region growing method to find a piece of region that connects the two vertices. We design two parameters in our region growing method to comply with the aforementioned criteria. Generally, given a drawing result, which is an image *I* with a resolution of M×N, we first locate the pixel positions of the two vertices in the image, and then use Algorithm 1 to find the number of paths between the two vertices. Assume the start pixel is $P_{s}$ and the target pixel is $P_{t}$. We specify a color threshold *C* and an angle threshold *L*. The target region *R* contains only $P_{s}$ initially. We start from the current pixel $P_{c}=P_{s}$, and search through all the neighboring pixels $\left\{P_{n}\right\}$ in a $W_{1}\times W_{1}$ window, where $W_{1}$ is the size of the window. We need to find all the neighboring pixels $\left\{P_{n}\right\}$ that meet three conditions: (1) the angle between the vector $\vec{P_{c}P_{t}}$ and the vector $\vec{P_{c}P_{n}}$ is not greater than *L*; (2) the angle between the vector $\vec{P_{s}P_{t}}$ and the vector $\vec{P_{c}P_{t}}$ is not greater than *L*; and (3) the Euclidean distance between the color of $P_{c}$ and the mean color $C_{m}$ of the region is not greater than *C*. Conditions (1) and (2) ensure that the region will be growing from the start vertex towards the target vertex with a specified angle limitation. *L* determines the sharpness of the paths in the region. Condition (3) simply ensures the color criterion. The qualified pixels that have not been visited are added to a candidate set. We then set the pixel that is closest to $C_{m}$ to be the new current point $P_{c}$ and add $P_{c}$ to the region *R*. The process continues until $P_{c}=P_{t}$ or the candidate set is empty. The region growing algorithm is illustrated in Algorithm 2.

**Figure 6:**
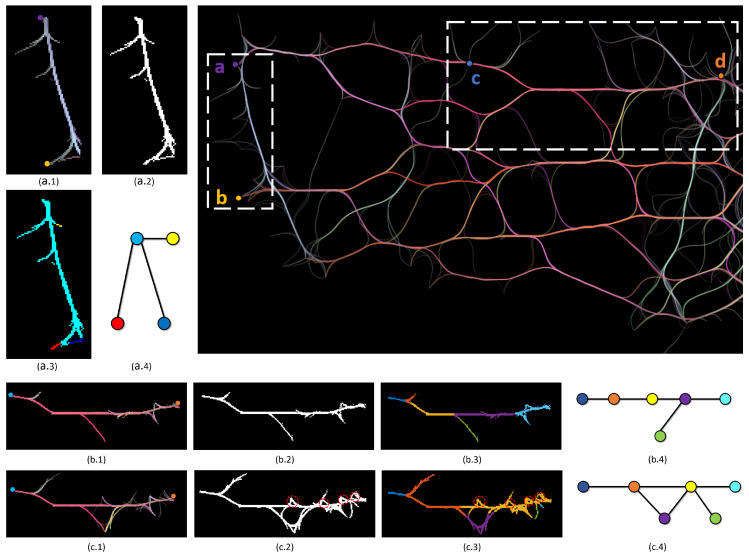
(**a.1**) The resulting region between two vertices *a* and *b* using Algorithm 2. (**a.2**) The input of Algorithm 3. (**a.3**) The output Algorithm 3. (**b.1**) The resulting region between two vertices *c* and *d* using Algorithm 2, where the color threshold *C* and angle threshold *L* are set to be 100 and 90 respectively. (**b.2**) The input of Algorithm 3. (**b.3**) The output of Algorithm 3. (**c.1**) The resulting region between node *c* and *d* using Algorithm 2, where the color threshold *C* and angle threshold *L* are set to be 190 and 150 respectively. (**c.2**) The input of Algorithm 3. (**c.3**) The output of Algorithm 3. (**a.4**–**c.4**) The corresponding transform graphs of (**a.3**–**c.3**), respectively.

Figure 6a shows a magnified and highlighted area of a FFTEB visualization. We want to find the region connecting two vertices *a* and *b*. Figure 6a.1 is the output of Algorithm 2. It shows that the region between *a* and *b* can be perfectly extracted. Another more complex example is shown in Figure 6b.1,c.1 presenting the impact of the input parameters *C* and *L*. In the highlighted area, we want to find the region connecting the vertices *c* and *d*. Using C=100 and L=90, we get the result of Figure 6b.1. Using C=190 and L=150, we have the resulting region of Figure 6c.1. The difference is obvious. In Figure 6b.1, we do not have the big hole in the middle of the region because the input color threshold *C* and angle threshold *L* are relatively small. *C* controls the acceptable color difference between candidate pixels and the region, while *L* determines that the sharpness of a portion of the region. They are very important parameters in our algorithm. The window size $W_{1}=1$ or 2 (1 or 2 pixel(s)) can generate very good results. However, *C* and *L* may impact the grown region largely, as in Figure 6b.1,c.1. We further discuss *C* and *L* in a heuristic study in Section 4.

**Algorithm 1**FindAllPaths.1:// Initialization2:*P_s_* // The start pixel3:*P_t_* // The target pixel4:*W*_1_ // The size of sliding window for Algorithm 25:*W*_2_ // The size of sliding window for Algorithm 36:*C* // The color threshold7:*I* // The *M* × *N* image8:*R* // The growing region9:*K* // The clusters10:*P* // The number of paths11:*N* // The number of node in graph12:*VISITED*[*N*] // The flag array that indicates if vertices are visited13:Find the source pixel *P_s_* and target pixel *P_t_*.14:// Given *I*, *W*_1_ and *C*, use region growing to find the region *R* connects *P_s_* and *P_t_*15:*R* ← RegionGrowing(*I*, *P_s_*, *P_t_*, *W*_1_, *C*)16:// Given the region *R*, use mean shift to calculate the clusters *K*17:*K* ← MeanShift(*R*, *W*_2_)18:Find the number of vertices *N* based on the separate components of *K*.19:// Based on the clusters *K*, find the source region *R_s_* and the target region *R_t_*20*P* ← Depth-firstSearch(*P*, *K*, *R_s_*, *R_t_*, *VISITED*[*R_s_*])

**Algorithm 2**RegionGrowing(I:input_image; $P_{s}:input\_source\_pixel$; $P_{t}:input\_target\_pixel$; $W_{1}:input\_window\_size$; C:input_color_threshold; L:input_angle_threshold).1:Assign the color of *P_s_* to *C_m_*.2:*R* // The growing region3:*C_m_* // The mean color of the growing region4:*P_c_* ← *P_s_* // Assign the source pixel to be the current pixel5:*S* ← ∅ // Initialize the candidates set6:Push *P_c_* into *R*.7:**while***P_c_*! = *P_t_* or *S*! = ∅ **do**8:  **for** each neighboring pixel *P_n_* of *P_c_* using the window size *W*_1_
**do**9:    **if** the angle *θ*_1_ between $\vec{P_{c}P_{t}}$ and $\vec{P_{c}P_{n}}$ <= *L*
**and** the angle *θ*_2_ between $\vec{P_{s}P_{t}}$ and $\vec{P_{c}P_{t}}$ <= *L*
**and** the color of *P_c_* − *C_m_* <= *C*
**then**10:      Push *P_n_* into *S*.11:    **end if**12:  **end for**13:  // Compute the next *P_c_*14:  Compute the pixel in *S* whose color is closest to *C_m_*, and assign the pixel to *P_c_*.15:  Compute the mean color of *S*, and assign the mean color to *C_m_*.16:  Pop *P_c_* from *S*.17:  Push *P_c_* into *R*.18:
**end while**
19:**return***R*.

After the region *R* is gained, we can consider how to find the number of paths between two pixels. Here, the problem is typically a graph problem that finding the number of paths between a source vertex and a target vertex in a graph where every pixel in *R* can be modeled as a vertex, and the connectivity of pixels can be modeled as edges. However, estimating the number of source-to-target paths in a graph is #P-complete [78]. To approximate the number of source-to-target paths, we could use depth-first search to enumerate all the unique paths from a source to a target, or a dynamic programming to statistically calculate the total number of unique paths from a source to a target. However, in our experiment, we found out that simply using the above methods is problematic in the applications of edge bundling visualization. For example, in Figure 6a.1, intuitively, there should be one path connecting *a* and *b*. Appendix A shows that a simple depth-first search will generate an incorrect (significantly large) number of paths for Figure 6a.1. Additionally, simply modeling every pixel as a vertex will make the computation time-consuming since a resulting region could consist of a considerable number of pixels. Appendix A also shows that dynamic programming is not appropriate to solve this problem in the applications of edge bundling visualization. Another problem is that neither the simple depth-first search nor dynamic programming cannot solve the problem that the small holes in the generated region, which is illustrated in Figure 6c.2. For instance, in Figure 6c.2, intuitively, there is only one path from *c* to *d*, whereas the small holes can generate unnecessary loop paths, which should be addressed.

We propose to use a simple mean shift to cluster the resulting region, then model the clusters into vertices, and conduct a modified depth-first search to approximately find the number of paths between two vertices in a region *R*. After the region *R* is found, we check that if the two vertices are in the region. If so, we use mean shift to cluster the region *R*. Otherwise, we conclude there is no path between the two vertices. The basic idea is that we first use mean shift to cluster *R* into distinct regions. Second, we construct a transform graph *T* that shows the connectivity of the distinct regions. We define the source region $R_{s}$ containing the pixel $P_{s}$, and the target region $R_{t}$ containing the pixel $P_{t}$. Finally, we use a modified depth-first search to calculate the number of paths between $R_{s}$ and $R_{t}$ in *T*.

The mean shift algorithm takes *R* as input. For each pixel, we define a window with a size $W_{2}$ around it and compute the mean of the pixels that have some color other than the background color. Then, we shift the center of window to the mean and assign the new position to the current pixel. We repeat this process until all pixels converge or the iterations exceed a certain amount of times. The simple mean shift algorithm is illustrated in Algorithm 3. Then, we consider the non-connected regions as distinct clusters. The distinct clusters can be considered as a graph *T*, where each cluster can be considered as a vertex in *T*. The connectivity of vertices in *T* is determined by the connectivity of the pixels. For example, if two pixels from two different clusters are neighbors, the two clusters have an edge. The output results of Algorithm 3 is demonstrated in Figure 6a.3–c.3. Figure 6a.4–c.4 shows the corresponding transform graphs of Figure 6a.3–c.3, respectively. In the three graphs, different colors mean distinct cluster labels. The results in Figure 6a.4–c.4 avoid the problem in Appendix A. In Figure 6b.3,c.3, the small hole problem is also addressed, where the hole is too small to be considered a branch or another path. However, if a hole is big enough, it can be considered as a branch or path, which is shown in Figure 6c.3. The window size $W_{2}$ determines the acceptable hole threshold. We find that it should be set to only 1 or 2 pixel(s), and Figure 6b.3,c.3 demonstrates the results.

**Algorithm 3**MeanShift(R:input_region; $W_{2}:input\_window\_size$).1:*K* // the cluster result2:*P_c_* // The position of the current pixel3:*S* // The temporal set4:*ITR* // The iteration number5:*STOP* // The flag that indicates all pixels do not move in the last iteration6:*STOP* ← False7:**while***ITR* < 300 and *STOP* = *False*
**do**8:  **for** each pixel *P_c_* of *R*
**do**9:    *S* ← ∅10:    **for** each neighboring pixel *P_n_* of *P_c_* using the window size *W*_2_
**do**11:      **if** the color of *P_c_* does not equal to the background color **then**12:        Push *P_n_* into *S*.13:      **end if**14:    **end for**15:    Compute the new position for *P_c_* based on *S*.16:  **end for**17:  // Check if some of the pixels have new positions18:  **if** none of the pixels in *R* moves **then**
*STOP* ← *True*19:  **end if**20:
**end while**
21:Give every separate component a distinct number, and assign the result to *K*.22**return***K*.

Finally, a modified depth-first search algorithm is used to count all possible paths between $R_{s}$ and $R_{t}$ in *T*. In this algorithm, it first sets the flag of every vertex to be unvisited. The algorithm starts from the source region $R_{s}$, and find the adjacent regions in a depth-first search manner until it reaches the target region $R_{t}$. Every time the algorithm reaches a new region, it sets the flag of the region to be visited. Hence, it will not form a loop path in *T*. If $R_{t}$ is reached, the counter of all possible paths increments one. The modification from the traditional depth-first search algorithm is that after a region is visited, we reset the flag of the current region to be unvisited, making this region available to other paths. Finally, if all other regions are visited, the algorithm ends. The modified depth-first search is illustrated in Algorithm 4.

**Algorithm 4**Depth-firstSearch(P:(GLOBAL)input_path_number; K:input_clusters; $R_{c}:input\_current\_region$; $R_{t}:input\_target\_region$), VISITED[]:input_source_flag.1:*P* // The number of path between *R_s_* and *R_t_*2:*VISITED*[*R_c_*] ← True3:**if***R_c_* = *R_t_*
**then**4:  *P* ← *P* + 15:
**else**
6:  **for** each adjacent region *R_n_* of *R_c_*
**do**7:    **if**
*VISITED*[*R_n_*] = False **then**
Depth-firstSearch(*P*, *K*, *R_n_*, *R_t_*, *VISITED*[*R_n_*])8:    **end if**9:  **end for**10:
**end if**
11:*VISITED*[*R_c_*] ← False

## 4. Application Examples

We provide several examples to show how to use the proposed metric and approximation method to evaluate and compare edge bundling algorithms. The edge bundling algorithms we used in this section include the force-directed edge bundling (FDEB) [33], the fast fourier transform edge bundling (FFTEB) [47], and the moving least squares edge bundling (MLSEB) [48]. The three methods cover three different edge bundling frameworks: force-directed, kernel estimation, and curve approximation. The three frameworks cover most of the edge bundling methods. Although only three methods are chosen to compare in this paper, our metric and method can be easily extended to other edge bundling methods. First, we briefly revisit the algorithms of FDEB, FFTEB, and MLSEB, respectively. Second, we conduct a heuristic study to discuss the color and angle thresholds in our path-finding method. Third, we show an example to use the proposed information-theoretic metric and path-finding method by exploring the parameters of the three edge bundling methods on a dataset. We discuss how users of edge bundling applications should choose the appropriate parameters of the edge bundling methods for their applications based on the analysis of the corresponding uncertainty H(W) and coverage λ. Fourth, we use different datasets to illustrate the similarities and differences among the three different edge bundling methods based on the analysis of the uncertainty H(W) and coverage λ and the corresponding visualizations.

In the following results, we render all edge bundling drawings into 800×800 images. The background color of these drawings is set to be (0, 0, 0, 0) in terms of RGBA value. We use a color-encoded method [46] in all renderings. The color-encoded method encodes the direction and the length of the corresponding edge with HSVA value (i.e., hue *H*, saturation *S*, value *V*, and alpha *A*). Using this method, short edges are better visible, and at the same time long edges can also attract attention. Additionally, since the value of H(W) can be large for some large graphs, we multiply H(W) with a factor $\frac{1}{p}$ for a better displaying (i.e., $H\left(W\right)=H\left(W\right)\times \frac{1}{p}$), where *p* is the number of all pairs of different nodes in the corresponding graph.

### 4.1. Revisit FDEB, FFTEB, and MLSEB

#### 4.1.1. FDEB

**Figure 7:**
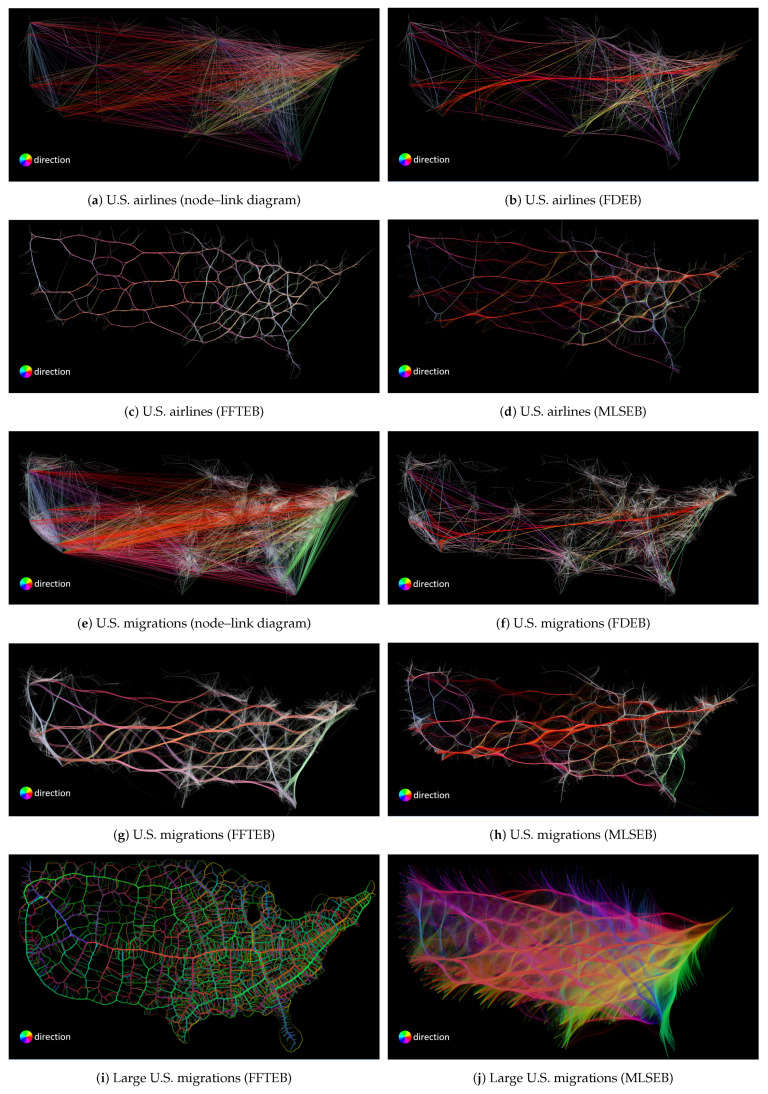
(**a**) node–link diagram of the U.S. airlines dataset. (**b**) FDEB visualization of the U.S. airlines dataset. (**c**) FFTEB visualization of the U.S. airlines dataset. (**d**) MLSEB visualization of the U.S. airlines dataset. (**e**) node–link diagram of the U.S. migrations dataset. (**f**) FDEB visualization of the U.S. migrations dataset. (**g**) FFTEB visualization of the U.S. migrations dataset. (**h**) MLSEB visualization of the U.S. migrations dataset. (**i**) FFTEB visualization of the large U.S. migrations dataset and (**j**) MLSEB visualization of the large U.S. migrations dataset.

FDEB uses a spring model to bundle edges in a graph drawing. It first employs similarity, namely compatibility measures to find the similar edges in a graph based on four spacial features (i.e., direction, length, position, and projection). The result of the compatibility measures is a soft clustering, which means one edge may belong to multiple groups. In every iteration of the bundling process, edges are subdivided into sample points. In the subdivision process, every edge is subdivided into identical number of sample points. A spring model is then applied in the subdivision points of edges to advect the edges in an iterative manner. One edge only interacts with its compatible edges. Hence, edges are bundled in a group manner, as shown in Figure 7b. The final image is generated when the spring system reaches an equilibrium state. One unique parameter in FDEB is the compatibility threshold used to determine how similar two edges are. Hence, it also determines the clustering results and the final edge bundling drawing.

#### 4.1.2. FFTEB

FFTEB uses fast fourier transform to accelerate large-graph bundling process. The basic idea of FFTEB is from the kernel density estimation edge bundling (KDEEB) [44]. KDEEB uses a uniform sampling method, where edges with different lengths have different numbers of sample points. KDEEB transforms an input graph into a density map using kernel density estimation, and then moves the sample points of edges towards the local density maxima to form bundles. The sample points of a single edge could belong to different bundles since the sample points are advected locally. FFTEB is an enhanced method of KDEEB. It shifts the bundling process from the image space to the spectral (frequency) space, thereby increasing computational speed. They address the performance issue by using GPU processing. An important parameter used in FFTEB is the window size of the kernel density estimation. The window size of the kernel density estimation determines the location of local density maxima in the drawing.

#### 4.1.3. MLSEB

MLSEB generates bundles based on moving least squares (MLS) approximation. The basic idea of MLSEB comes from reconstructing a smooth curve from an unorganized point cloud. It first discretizes all edges into a set of sample points. Then, it uses moving least squares approximation to reconstruct a smooth curve based on locally close sample points with a window size. The close sample points of edges will be projected onto the local regression curve iteratively. After each iteration, the sample points of different paths locally gather closer. Using the advected sample points of each path as control points, a B-spline curve is generated for each edge. The curve-like bundles are thus formed. The moving least squares method also gives a result that one edge may belong to different bundles, as shown in Figure 7d. MLSEB also uses the same uniform sampling for each edge as FFTEB. The important parameter is the compact support (i.e., the window size) of the moving least squares approximation.

### 4.2. Heuristic Study

In this section, we conduct a heuristic study to discuss the color and angle thresholds in our proposed method for different datasets. The datasets we used in this section are the U.S. airlines dataset with 2100 edges and 235 vertices, and the U.S. migrations dataset with 9780 edges and 1700 vertices.

Since we employ the algorithms in Section 3.4 to mimic an observer’s perception to track paths between two vertices in a graph drawing, we conduct a heuristic study to discuss the parameters, the color threshold *C* and the angle threshold *L*. The rationale of this heuristic study is to choose the optimal thresholds for our evaluation algorithm. In the heuristic study, we compare the coverage and the uncertainty of the U.S. airlines using Figure 7b–d, as well as the U.S. migrations data using Figure 7f–h with different values of color threshold and angle threshold. The values of color threshold we used are 100, 120, 150, 170, and 190. The values of angle threshold we used are $45^{\circ },\;90^{\circ }\;\mathrm{and}\;135^{\circ }$. The compatibility of the FDEB drawings in Figure 7b,f, is 0.7. The kernel size of the FFTEB drawings in Figure 7c,g,i is 5%. The compact support of the MLSEB drawings in Figure 7d,h,j is 5%. In Table 1, we list the coverage and uncertainty values for the corresponding combination of color and angle thresholds. c100_a45 in the table means $C=100^{\circ }$ and $L=45^{\circ }$, etc. In Table 1, we can see that, as *C* and *L* increase, the coverage and uncertainty increase, which matches our expectation that higher *C* and *L* will give a higher coverage and uncertainty. One important point is that, if we use the same color threshold and different angle thresholds, the corresponding gradients of coverage and uncertainty between $L=90^{\circ }$ and $L=135^{\circ }$ are significantly smaller than the gradients of $L=45^{\circ }$ and $L=90^{\circ }$ for all results. For example, for FFTEB, the coverage and uncertainty differences between c100_a45 and c100_a90 are significantly smaller than the differences between c100_a90 and c100_a135. We find that this phenomenon exists for all edge bundling methods we tested. Hence, we conclude that $90^{\circ }$ can be used as an appropriate angle threshold. Choosing $90^{\circ }$ as the angle threshold can ensure the path in the growing region will not turn abruptly. One example between small and large angle thresholds are shown in Figure 6b.1,c.1. On the other hand, for the color threshold, we can know that, when the color threshold reaches 190, the coverage of all methods become almost 1. Therefore, in the following comparison, we list different color thresholds smaller than 190 with the same angle threshold $L=90^{\circ }$. Since we do not have an optimal color threshold, we compute the values of coverage and uncertainty using different color thresholds. We compute the average sum of all the color thresholds of the corresponding coverage and uncertainty in order to obtain succinct statistic results.

### 4.3. Comparison I

In this section, we evaluate the coverage and uncertainty of the three methods with different parameters. We use an example to show how to use the proposed metric and approximation method to choose an appropriate parameter for a specific edge bundling application. The dataset we use in this section is the U.S. airlines. To explore the parameter combinations of edge bundling methods is a tremendous work since many parameters and factors can be taken into account, such as the color-encoded method, the number of sampling points of every edge, the unique parameter of a method, and so on. These parameter combinations vary from case to case. It is not feasible to list many parameter combinations in a single paper. Hence, we rather use an simple instance to illustrate how users can employ our proposed metric and path-finding method to choose parameters for edge bundling applications. Recall that, in Section 4.1, we mentioned the unique and important parameters for the three methods respectively, which are the compatibility threshold for FDEB, the kernel size for FFTEB, and the compact support for MLSEB. We use a bivariate analysis to analyze the results of the above methods with their different corresponding parameters. We focus on analyzing these parameters in this paper. Our analysis can be easily extended to more complex parameter configurations for other edge bundling methods.

**Figure 8:**
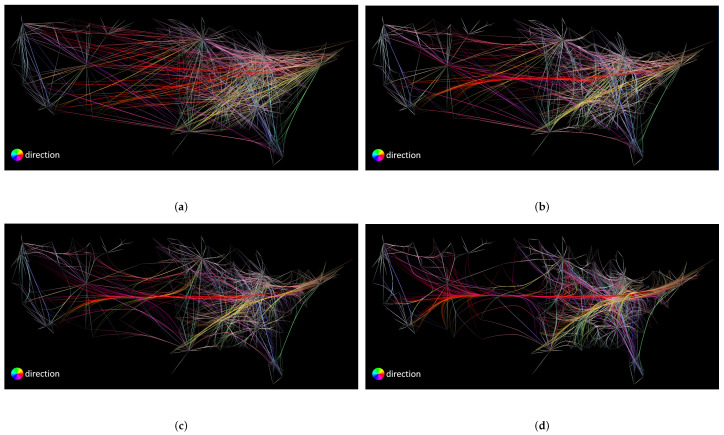
FDEB drawings of the U.S. airlines using different compatibility threshold values: (**a**) 0.8; (**b**) 0.7; (**c**) 0.6; and (**d**) 0.5.

**Figure 9:**
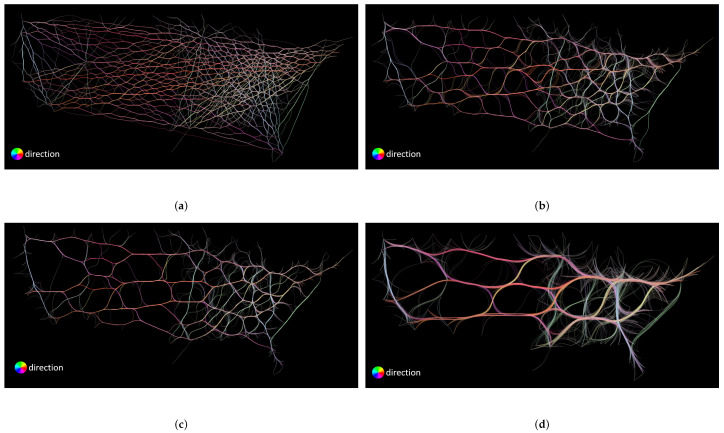
FFTEB drawings of the U.S. airlines using different kernel threshold values: (**a**) 1% kernel size; (**b**) 3% kernel size; (**c**) 5% kernel size; and (**d**) 10% kernel size.

**Figure 10:**
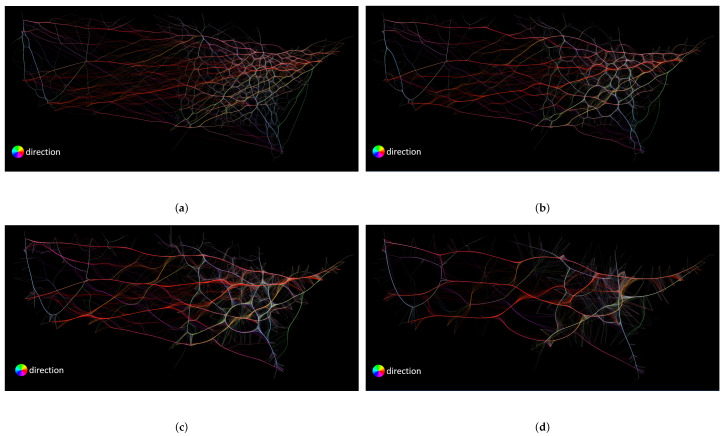
MLSEB drawings of the U.S. airlines using different compact support values: (**a**) 1% compact support; (**b**) 3% compact support; (**c**) 5% compact support; and (**d**) 10% compact support.

First, we discuss the parameters and configurations used in this comparison. We use four different compatibility thresholds for FDEB. Remember that the compatibility threshold of FDEB corresponds to the soft clustering result that determines the final graph layout. In Figure 8a–d, we set the compatibility thresholds to be 0.8, 0.7, 0.6, and 0.5, respectively. For FFTEB, the most important parameter is the kernel size of the density estimation, which determines how the sample points of edges coverage. We set the kernel size of FFTEB to be 1%, 3%, 5%, and 10% of the image size, as shown in Figure 9. For MLSEB, the most significant parameter is the compact support of the moving least squares approximation. It is essentially a window size to estimate the weights of neighboring sample points that are used to approximate a local regression curve. We set the compact support of MLSEB to be 1%, 3%, 5% and 10% of the image size, which are shown in Figure 10.

**Figure 11:**
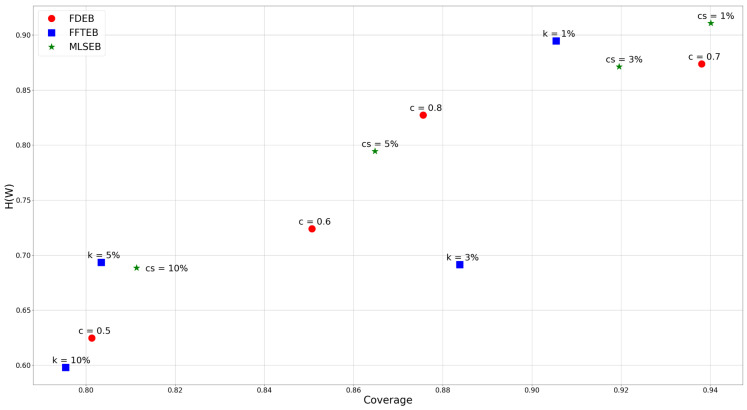
The bivariate analysis of FDEB, FFTEB, and MLSEB using different parameters.

The angle and color thresholds we used in this comparison are L=90 and *C* = 100, 110, 120, 130, 140, 150, 160, 170, 180, and 190, respectively. As mentioned before, to show a succinct result, we compute the average sum of all the color and angle threshold combinations of the corresponding coverage and uncertainty respectively. Figure 11 shows a scatter plot of coverage versus uncertainty of different parameters of the three methods. We first analyze the impact of compatibility threshold to the FDEB drawing. The label c=0.6 means the compatibility is set to be 0.6 for FDEB, etc. The compatibility threshold determines the clustering result. As the compatibility threshold decreases, the similarity estimation is more loose, which results in more edges are bundled together. We can obverse this trend in Figure 8. In Figure 11, the coverage and conditional entropy increase monotonically with the increase of the compatibility value, which matches our expectation that if more edges are bundled, it becomes less uncertain in perception but hard to reconstruct the original graph structure through perception. This phenomenon also happens to MLSEB in this dataset. As the compact support (i.e., the window size) increases, edges are merged into larger bundles. Meanwhile, the coverage and conditional entropy also decrease accordingly. We also look at the kernel size of FFTEB. As the kernel size increases, the edges are bundled tighter, and more edges gather into larger bundles. Greater kernel size results in less coverage and conditional entropy. However, we are surprised to see that, when k=3% and k=5%, the conditional entropy of the two results are almost the same, but k=3% shows a significantly greater coverage. By comparing Figure 9b,c, we find that the visual result of k=3% is very similar to the result of k=5%. As mentioned in Section 3.3, we should achieve a high coverage and low conditional entropy in an edge bundling drawing. Hence, Figure 9b is better than Figure 9c in terms of the proposed coverage and conditional entropy.

Analyzing the coverage and conditional entropy can help users choose optimal parameters for the edge bundling applications. We can set a 2D interval to select the optimal parameter for our desire edge bundling methods. Since we intend to achieve high coverage and low conditional entropy in an edge bundling drawing, we can define a 2D interval by setting a lower bound for coverage and an upper bound for conditional entropy respectively. Take Figure 11 as an example; we can set the lower bounds of coverage to be 0.85 and the upper bound of conditional entropy to be 0.8, which means we want the method can achieve at least 85% coverage and at most 80% conditional entropy. The 2D interval gives three instances that are a FDEB with a 0.6 compatibility threshold, a FFTEB with a 3% kernel size, and an MLSEB with a 5% compact support. In this analysis, users can determine a customized 2D interval to select the appropriate parameters for their desire edge bundling applications. This analysis can also be easily extended to cases with more complex parameter combinations.

### 4.4. Comparison II

**Figure 12:**
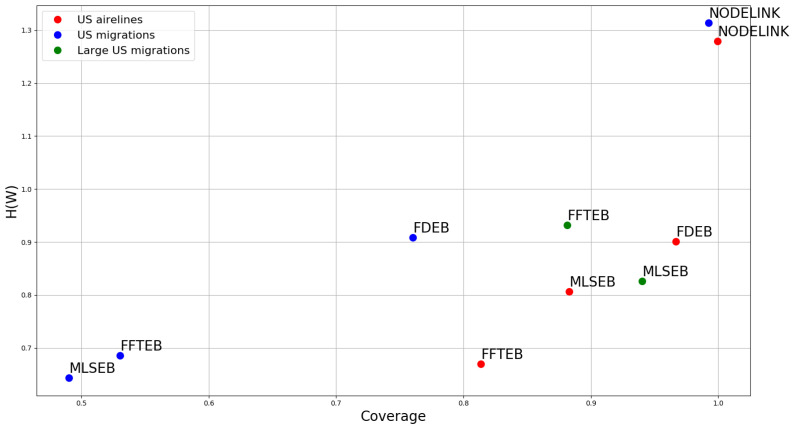
The bivariate analysis of FDEB, FFTEB, and MLSEB using different datasets.

The purpose of this section is not to merely compare the quality of the three methods using different datasets. We have introduced a bivariate analysis to analyze and compare the three methods with different parameters for one specific application. Users and readers should follow the analysis method in Section 4.3 to test different parameter combinations for different datasets. In this section, we discuss the three edge bundling methods in depth. The main focus of this section is on analyzing their similarity and difference based on the visual result, coverage, and conditional entropy across different datasets. We use three datasets: (1) U.S. airlines (2100 edges and 235 nodes); (2) U.S. migrations (9780 edges and 1700 nodes); and (3) large U.S. migrations (545,881 edges and 3075 nodes). We use segment-based node–link diagram, FDEB, FFTEB and MLSEB to visualize the three datasets, and compute the corresponding coverage and conditional entropy values. Due to the limitation of the implementation of FDEB, we cannot construct an edge bundling drawing for large U.S. migration with FDEB. Remember that we used angle threshold L=90 and color threshold C=100, 110, 120, 130, 140, 150, 160, 170, 180, and 190 in our path-finding algorithm. Moreover, we compute the average sum of all angle and color thresholds for coverage and conditional entropy, respectively. The additional images we used in this section are shown in Figure 7. We plot the corresponding scatter plot of H(W) and λ in Figure 12.

First, we analyze the difference between the node–link diagram drawing and the three edge bundling methods. The node–link diagrams in Figure 7a,e have relatively more visual clutter than other edge bundling methods because of edge crossings and total area used. The visual clutter can be quantitatively interpreted by our proposed conditional entropy H(W). The coverage and conditional entropy of the node–link diagram drawings fully reflect this phenomenon. According to the result of Figure 12, the node–link diagram has overall the highest coverage; however, its uncertainty H(W) is significantly larger than the other three edge bundling methods, which matches our expectation. The coverage of the node–link diagram is saturated but the visual result is of high uncertain, which can be interpreted by H(W). Hence, we have an impression that, although node–link diagram gives the highest coverage rate, it is also the most uncertain visualization compared to the other three edge bundling methods.

Second, we observe that some results of Figure 9 and Figure 10 are visually similar. However, these results are very different from FDEB in Figure 8. As we revisited the FDEB in Section 4.1, the final result of FDEB is determined by the edge clustering result. Since only compatible edges will be bundled together, the clustering result determines the potential position where the edges are in the final drawing. The clustering result is based on the edge level. In FFTEB, the sample points of edges are moved to the local density maxima according to the gradients of the local density estimation. It uses a kernel size to determine the effect of the local density estimation. In MLSEB, bundled curves are locally approximated by the local compact support in a regression manner. To construct such a bundled curve, it also needs to use a compact support (window size) to estimate the weights and positions of the local neighboring sample points. Indeed, both FFTEB and MLSEB use a window size (i.e., kernel and compact support, respectively) in their bundling processes. The estimation process of both methods is essentially density estimation. Hence, the clustering or merging process of FFTEB and MLSEB is based on the sample point level. An interesting observation is that, in U.S. airlines and U.S. migrations datasets, FDEB has the highest coverage and conditional entropy value, and the coverage and conditional entropy are significantly greater than the other two edge bundling methods. Another interesting observation is that, when the compatibility threshold is high (c=0.8), the result in Figure 8a resembles Figure 7a. Hence, it yields great values of coverage and conditional entropy. Based on the above observation, we can conclude that edge clustering methods prone to generate a more visual uncertain edge bundling result. Although it reduces small-angle edge crossings, it cannot reduce the edge crossings among edge clusters. This is why FDEB results visually have more edge crossings than the other two methods, which can be reflected by the condition entropy H(W) in Figure 12.

As the density estimations of FFTEB and MLSEB are similar, some results of FFTEB and MLSEB are similar as well (e.g., Figure 9a versus Figure 10a, and Figure 9b versus Figure 10b). FFTEB and MLSEB both produce web-like bundle effect that is completely different from FDEB. What differentiates them is the advection methods of the sample points. FFTEB uses the gradients of the density estimation as the advection vector while MLSEB uses regression method to project the sample points to new positions. When the kernel size and compact support are both below 5%, their results are almost identical. However, when the kernel size and compact support reach 5% or greater, their results become different. Figure 7c,d,g–j illustrates this phenomenon. When we use a very large dataset, the large U.S. migrations (545,881 edges and 3075 nodes), their results become completely different, as shown in Figure 7i,j. The resulting scatter plot in Figure 12 shows that FFTEB and MLSEB have their own advantages and disadvantages for U.S. airlines and U.S. migrations. However, MLSEB outperforms FFTEB in the very large dataset with respect to both coverage and H(W).

## 5. Conclusions and Future Work

In this paper, we present a new information-theoretic metric for evaluating the overall uncertainty of edge bundling visualizations. We used the theory of the mutual information to assess the final results of edge bundling methods. Based on the information-theoretic pipeline of Chen et al. [8,9], we have discussed the benefits and drawbacks of edge bundling visualizations. We defined and quantified the uncertainty H(X|Y) of the visual description of the relation between two vertices. We attributed H(X|Y) to the actual number of paths between the two vertices in the corresponding drawing. H(X|Y) basically measures the length of the bits necessary to describe the relation given the visual description. We then argued that the average of total sum of H(X|Y) of all pairs of vertices can be used to evaluate the uncertainty of edge bundling visualizations, i.e., H(W). The key idea of our evaluation is that the lower the value of H(W) produced by an edge bundling method, the better the method is. We show an example for users to choose optimal parameters for their desired edge bundling applications. We used this metric to compare three different edge bundling methods, the force-directed edge bundling (FDEB), the fast fourier transform edge bundling (FFTEB), and the moving least squares edge bundling (MLSEB) in depth, which has not been fully discussed in the existing literature. We found that H(W) can correctly reflect the degree of ambiguity of edge bundling algorithms. Note that our framework only focuses on the vis-encoder process of Chen et al.’s pipeline. We admit that some factors in the vis-channel and vis-decoder process, such as display resolution, view angle, interaction, and cognition would affect the final observer’s comprehension. We focus on the vis-encoder process because it is easier to explain the concept of the uncertainty caused by edge bundling visualizations. Although we did not take these factors into account, our methodology is easy to extend in the vis-channel and vis-decoder process. In addition to the path-finding method, we will try to use other methods to approximate the value of coverage and conditional entropy of edge bundling methods. We also would like to include user studies in our future work. A user study can testify and give more accurate values of coverage and conditional entropy from a user perception perspective. In addition, general graph visualization can use our information-theoretic metric to quantify the quality of their visualization results. Our proposed metric can also be used to generate novel graph visualizations and be applied to real-world applications.

## Figures and Tables

**Table 1:** A heuristic study to choose color and angle thresholds.

	FDEB				FFTEB				MLSEB			
	U.S. Airlines		U.S. Migrations		U.S. Airlines		U.S. Migrations		U.S. Airlines		U.S. Migrations	
Configuration	H(W)	λ	H(W)	λ	H(W)	λ	H(W)	λ	H(W)	λ	H(W)	λ
c100_a45	0.661	0.795	0.791	0.669	0.225	0.437	0.457	0.268	0.486	0.584	0.349	0.258
c100_a90	0.751	0.817	0.856	0.688	0.319	0.554	0.508	0.359	0.607	0.679	0.380	0.307
c100_a135	0.793	0.830	0.870	0.699	0.343	0.575	0.518	0.389	0.646	0.681	0.391	0.339
c120_a45	0.838	0.944	0.846	0.671	0.326	0.564	0.527	0.336	0.594	0.742	0.469	0.288
c120_a90	0.907	0.972	0.888	0.705	0.450	0.689	0.577	0.397	0.710	0.810	0.513	0.346
c120_a135	0.945	0.975	0.893	0.713	0.485	0.709	0.588	0.421	0.753	0.827	0.529	0.363
c150_a45	0.893	0.988	0.873	0.763	0.642	0.770	0.681	0.459	0.815	0.947	0.680	0.398
c150_a90	0.926	0.997	0.911	0.786	0.819	0.867	0.723	0.521	0.889	0.982	0.718	0.458
c150_a135	0.961	0.997	0.928	0.792	0.864	0.877	0.740	0.558	0.925	0.985	0.726	0.467
c170_a45	0.909	0.996	0.883	0.800	0.735	0.911	0.740	0.582	0.845	0.966	0.702	0.673
c170_a90	0.934	0.998	0.940	0.822	0.848	0.973	0.782	0.691	0.902	0.988	0.746	0.702
c170_a135	0.965	0.998	0.952	0.831	0.894	0.978	0.800	0.729	0.934	0.990	0.760	0.719
c190_a45	0.922	0.999	0.938	0.809	0.759	0.937	0.857	0.692	0.858	0.977	0.726	0.682
c190_a90	0.945	0.999	0.954	0.837	0.870	0.988	0.889	0.776	0.905	0.995	0.761	0.703
c190_a135	0.976	0.999	0.967	0.849	0.920	0.995	0.901	0.792	0.936	0.995	0.789	0.718

**Figure A1:**
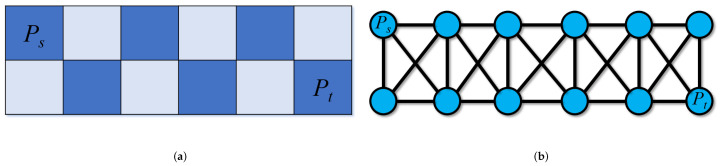
An example to approximate the number of paths between a source pixel $P_{s}$ and a target pixel $P_{t}$: (**a**) the connecting region between $P_{s}$ and $P_{t}$; and (**b**) the transform graph.

## Abbreviations

The following abbreviations are used in this manuscript:

FDEB	force-directed edge bundling
FFTEB	fast fourier transform edge bundling
MLSEB	moving least squares edge bundling

## Appendix A Estimating the Number of Source–Target Paths

Figure A1 shows an example to approximate the number of paths between a source pixel $P_{s}$ and a target pixel $P_{t}$. Every square in Figure A1a represents a pixel. All pixels in Figure A1a show a connecting region. To approximate the number of paths between the source pixel and the target pixel, we can transform Figure A1a into a graph, as shown in Figure A1b. We limit the traverse direction of every pixel from going back in order to avoid loop paths. Traversal methods, such as depth-first search, can be applied in order to enumerate the unique paths between the two pixels. However, the traversal method will generate far more number of unique paths. However, visually. there is only one path between the two pixels in Figure A1a, as the region can be considered as a path as a whole. Similarly, the traditional dynamic programming method cannot solve this problem either. Assume we define a subproblem as how many source-sink paths are through the current pixel. The dynamic programming method is not effective because the subproblem is not solved only once. Hence, depth-first search and dynamic programming cannot appropriately solve this type of problem.
